# Artificial Intelligence in the Management of Hereditary and Acquired Hemophilia: From Genomics to Treatment Optimization

**DOI:** 10.3390/ijms26136100

**Published:** 2025-06-25

**Authors:** Laura Giordano, Antonio Gaetano Pagana, Paola Lucia Minciullo, Manlio Fazio, Fabio Stagno, Sebastiano Gangemi, Sara Genovese, Alessandro Allegra

**Affiliations:** 1 Division of Hematology, Department of Human Pathology in Adulthood and Childhood “Gaetano Barresi”, University of Messina, Via Consolare Valeria, 98125 Messina, Italy; laura.giordano@polime.it (L.G.); antoniopagana94@gmail.com (A.G.P.); manliofazio@hotmail.it (M.F.); aallegra@unime.it (A.A.); 2 Allergy and Clinical Immunology Unit, Department of Clinical and Experimental Medicine, University of Messina, Via Consolare Valeria, 98125 Messina, Italy; gangemis@unime.it; 3 Institute for Biomedical Research and Innovation (IRIB), National Research Council of Italy (CNR), 98164 Messina, Italy; sara.genovese@irib.cnr.it

**Keywords:** hereditary hemophilia, acquired hemophilia, genetic mutation, molecular analyses, coagulation factors, artificial intelligence, machine learning, bleeding risk, precision medicine

## Abstract

Hemophilia, an X-linked bleeding disorder, is characterized by a deficiency in coagulation factors. It manifests as spontaneous bleeding, leading to severe complications if not properly managed. In contrast, acquired hemophilia is an autoimmune condition marked by the development of inhibitory antibodies against coagulation factors. Both forms present significant diagnostic and therapeutic challenges, highlighting the need for advanced genetic, molecular, laboratory, and clinical assessments. Recent advances in artificial intelligence have opened new avenues for the management of hemophilia. Machine learning and deep learning technologies enhance the ability to predict bleeding risks, optimize treatment regimens, and monitor disease progression with greater precision. Artificial intelligence-driven applications in medical imaging have also improved the detection of joint damage and hemarthrosis, ensuring timely interventions and better clinical outcomes. Moreover, the integration of artificial intelligence into clinical practice holds the potential to transform hemophilia care through predictive analytics and personalized medicine, promising not only faster and more accurate diagnoses but also a reduction in long-term complications. However, ethical considerations and the need for data standardization remain critical for its widespread adoption. The application of artificial intelligence in hemophilia represents a paradigm shift towards precision medicine, with the promise of significantly improving patient outcomes and quality of life.

## 1. Introduction

### 1.1. General Considerations on Clinical Background of Hemophilia

The X-linked bleeding diseases hemophilia A and B are defined by a partial or total lack of coagulation factor VIII (FVIII) or IX (FIX). Hemophilia is categorized as mild, moderate, or severe based on residual plasma factor activity. Without prophylaxis, individuals with severe hemophilia (PwH) experience frequent spontaneous bleeding events (BEs) into joints and muscles. This lowers the patient’s quality of life by increasing the risk of medical and socioeconomic morbidity and necessitating substantial management efforts [1,2,3,4].

In contrast, acquired hemophilia, particularly acquired hemophilia, is an autoimmune condition where inhibitory antibodies target coagulation factors, often leading to severe bleeding in previously healthy individuals, predominantly in the elderly or postpartum women [5]. Both inherited and acquired hemophilia are still difficult to diagnose, requiring both extensive clinical assessment and advanced laboratory tests, including factor activity testing and inhibitor screening [6]. Management strategies have evolved considerably over recent decades, with advances such as recombinant clotting factors, bypassing agents, monoclonal antibodies like Emicizumab, and ongoing efforts in gene therapy [7,8].

Nevertheless, challenges persist in timely diagnosis, individualized treatment planning, prediction of inhibitor development, and optimal long-term monitoring [9]. Simultaneously, the field of artificial intelligence (AI) has experienced remarkable growth, offering new tools for analyzing complex biomedical data, identifying disease patterns, and enhancing diagnostic and therapeutic precision [10]. In rare diseases like hemophilia, where datasets are traditionally small and clinical heterogeneity is high, AI holds the potential to bridge critical gaps by leveraging machine learning (ML) algorithms, deep learning models, and natural language processing to support clinical decision making [11,12,13].

AI encompasses a broad spectrum of computational techniques aimed at simulating human cognitive functions such as reasoning, decision making, and perception. Within this domain, ML represents a specialized subset that focuses on enabling systems to learn from data and improve performance over time without explicit programming. In the medical field, AI is employed in clinical decision support systems, robotic surgery, and advanced imaging diagnostics, while ML is particularly effective in predictive analytics, patient stratification, and natural language processing of clinical records. In the context of hemophilia research, AI contributes to the optimization of gene therapy strategies, including vector design and off-target prediction, and supports telemedicine platforms for remote patient management. Conversely, ML techniques are instrumental in predicting clinical outcomes, classifying disease severity based on factor levels and patient history, and identifying novel biomarkers for early diagnosis and monitoring. These technologies collectively enhance the precision and personalization of care in hemophilia and other complex disorders.

### 1.2. Hemophilia A

Hemophilia A, characterized by a deficiency in coagulation factor VIII, represents the most common severe inherited bleeding disorder. It is an X-linked recessive disease that can result from point mutations, deletions, or the presence of premature stop codons. The application of restriction enzymes has enabled the identification of more than 80 mutations responsible for alterations in the FVIII gene [14]. The condition primarily affects males, while females may be carriers of a mutation on one of the two X chromosomes. Female carriers are typically asymptomatic but may exhibit mild bleeding tendencies due to lyonization (random X-chromosome inactivation).

**Figure 1:**
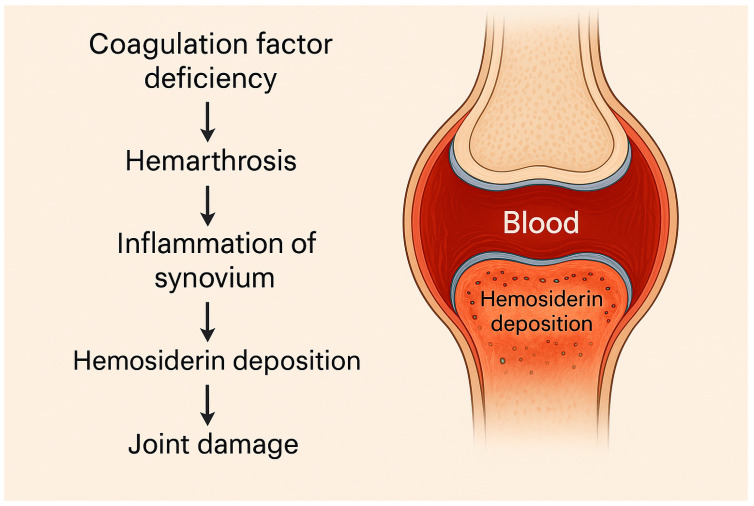
Pathogenesis of hemarthrosis in hemophilia.

The clinical manifestations of the disease are closely related to the level of factor VIII coagulant activity. In healthy individuals, FVIII activity ranges between 50% and 150%. Patients with <1% activity are classified as having severe hemophilia; activity levels between 1% and 5% define moderate hemophilia, and levels between 5% and 40% are indicative of mild hemophilia [15]. The main clinical features of hemophilia include hemarthrosis (bleeding into joints), particularly in large joints, presenting pain, swelling, and loss of joint function (Figure 1). The most serious complication is hemophilic arthropathy, as recurrent hemarthroses may lead to chronic synovitis, cartilage degeneration, and reactive changes in surrounding bone and soft tissue structures [16]. Hematomas involving subcutaneous connective tissue or muscle tissue may be superficial or deep and can occur spontaneously or following trauma. Pseudotumor is a rare complication in which recurrent hematomas expand and become encapsulated radiographically; these lesions may appear as mass-like structures that infiltrate surrounding tissues [17]. Mucosal bleeding commonly involves the oral, nasal, or gastrointestinal mucosa. Hematuria frequently occurs spontaneously in patients with severe hemophilia. Neurological complications may also occur due to intracranial hemorrhage [18,19,20].

### 1.3. Hemophilia B

Hemophilia B is an X-linked recessive hereditary bleeding disorder characterized by reduced activity of coagulation factor IX. Coagulation factor IX is a single-chain, vitamin K-dependent glycoprotein. Its activation can occur either via activated factor XI in the presence of calcium or through the complex formed by tissue factor, activated factor VII, and calcium. Once activated, factor IX is capable of activating factor X. The clinical severity of hemophilia B is correlated with the residual functional activity of factor IX. As in hemophilia A, the disorder is classified into severe forms when factor IX levels are below 1%, moderate forms when levels range between 1% and 5%, and mild forms when levels are between 5% and 40%. The clinical manifestations of hemophilia B are essentially indistinguishable from those of hemophilia A [14,15,21].

### 1.4. Diagnosis of Hereditary Hemophilia

Typically, in patients with a bleeding history suggestive of hemophilia, laboratory evaluation should include activated partial thromboplastin time (aPTT), prothrombin time (PT), and platelet count. In individuals with hemophilia, the platelet count and PT are within normal limits, whereas the aPTT is markedly prolonged. The diagnosis is confirmed by measuring factor VIII levels in hemophilia A and factor IX levels in hemophilia B. The differential diagnosis of hereditary hemophilia should include von Willebrand disease, in which the factor VIII deficiency is secondary to a qualitative or quantitative defect of its carrier protein (von Willebrand factor), as well as acquired hemophilia, which will be discussed later [4,22,23].

### 1.5. Acquired Hemophilia

Acquired hemophilia is a rare autoimmune disorder caused by the presence of immunoglobulin G (IgG) autoantibodies that inhibit a coagulation factor, most commonly factor VIII; this condition is therefore referred to as acquired hemophilia A (AHA) [24]. AHA primarily affects older adults, with a median age at diagnosis ranging from 64 to 78 years.

However, it can also occur in association with pregnancy and autoimmune diseases, particularly in younger individuals [25,26,27,28]. While most cases are idiopathic, AHA has also been linked to malignancies, autoimmune conditions (with rheumatoid arthritis being the most frequent), infections, dermatologic disorders, and certain medications, such as interferon alpha [29,30,31,32,33,34].

Pediatric cases are uncommon; one review identified 42 instances in children, 6 of which were attributed to the transplacental transfer of maternal antibodies [35,36,37,38]. Emerging evidence indicates that immune tolerance failure arises from a multifactorial interplay of genetic predisposition and environmental triggers. Although approximately 30% of patients do not require hemostatic therapy, the clinical severity of bleeding episodes varies considerably and can pose severe risks to life and safety. A recent study found that 94.6% of patients presented with bleeding, with 77% experiencing spontaneous bleeding and 70% classified as having severe bleeding (defined as hemoglobin [Hb] < 8 g/dL or a drop in Hb > 2 g/dL). Due to the non-linear, second-order kinetics of anti-FVIII antibodies, FVIII activity levels do not reliably predict bleeding risk; significant hemorrhage can occur even when FVIII activity is only moderately reduced. The most frequently reported site of bleeding is subcutaneous tissue (>80%), followed by muscular (>40%), gastrointestinal (>20%), and genitourinary, retroperitoneal, and other anatomical locations. The mortality rate for AHA is estimated at around 20%, particularly among individuals over 65 years of age and those with comorbid malignancies. While the primary cause of death is often the underlying disease (46%), in many cases (38%), the cause remains unidentified, likely due to limited long-term follow-up in available studies.

AHA should be considered in patients—especially older adults and women in the peripartum or postpartum period—who present with new-onset unexplained bleeding, an isolated prolongation of activated partial thromboplastin time (aPTT), and a normal prothrombin time (PT) [24,39]. Confirmatory testing includes measurement of factor VIII activity and inhibitor titration. However, the inhibitor titer determined by the Bethesda assay may not accurately reflect bleeding tendency, as these autoantibodies exhibit a non-linear pattern of neutralization and may result in incomplete inactivation of factor VIII even at high concentrations [40,41,42,43,44].

### 1.6. Management of Hemophilia

Treatment goals in hemophilia involve the prevention and control of bleeding, management of complications (such as inhibitor development), and preservation of joint function. For hereditary hemophilia, prophylactic factor replacement remains the standard; however, therapies such as Emicizumab—a bispecific antibody that bridges activated factor IX (FIXa) and factor X (FX) to restore the missing function of activated factor VIII (FVIIIa)—have transformed prophylaxis for Hemophilia A, especially in patients with inhibitors [45].

Gene therapy has shown promising results, offering the potential for sustained expression of clotting factors after a single infusion of adeno-associated viral (AAV) vectors carrying factor VIII or IX genes [4,22,23,46,47].

**Figure 2:**
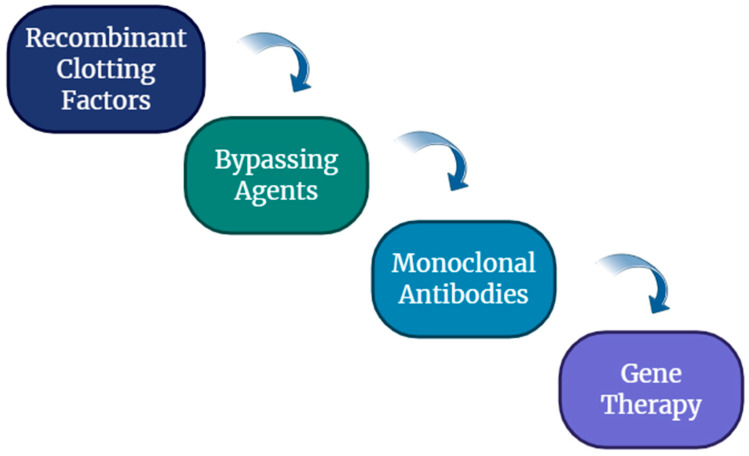
Modern therapies in hemophilia management.

In acquired hemophilia, bleeding is managed using bypassing agents such as activated prothrombin complex concentrate (FEIBA) and recombinant activated factor VII (rFVIIa, NovoSeven); more recently, recombinant porcine factor VIII has also been approved for use. Inhibitor eradication is achieved through immunosuppressive therapy, typically with corticosteroids—either alone or in combination with cyclophosphamide—or with the anti-CD20 monoclonal antibody [48,49,50,51,52,53] (Figure 2).

## 2. Focus on Artificial Intelligence

In recent years, AI has emerged as a revolutionary technology capable of transforming numerous aspects of everyday life and medicine. Platforms such as Siri, Alexa, Google Assistant, and ChatGPT have become common tools, able to interpret voice commands and respond intelligently to user requests. In industrial and commercial settings, machine learning and deep learning algorithms are employed to optimize production processes, enhance user experiences, and refine marketing strategies through the analysis of vast amounts of data. The impact of AI is particularly significant in the medical field, where it is redefining paradigms of diagnosis and treatment. In healthcare, AI is applied across various domains, including radiological image analysis, clinical data management, drug discovery, and cancer diagnostics. Deep learning systems have enabled the development of applications capable of analyzing pathological images with an accuracy that rivals, and sometimes surpasses, that of expert pathologists. For instance, the use of AI in diagnosing breast cancer and melanoma has demonstrated improvements in diagnostic precision and a reduction in human error [54,55].

Another concrete example is represented by the automation of medical coding and the management of clinical information. Natural language processing (NLP) technologies are employed to interpret and classify clinical documents, facilitating data management and the retrieval of relevant information [55]. In oncology, AI is used not only to identify tumors in radiological images but also to predict their evolution through machine learning models trained on extensive clinical databases [56].

Additionally, drug discovery has greatly benefited from AI, with deep learning algorithms enabling virtual screening of millions of chemical compounds, accelerating the research and development phase of new therapeutic treatments [57]. Among the most widely used platforms in the medical field are IBM Watson Health, which leverages AI algorithms to provide decision support to physicians, and PathAI, a system that uses deep learning to improve histopathological diagnoses. Google Health is also advancing technology for medical image interpretation, such as diagnosing diabetic retinopathy and lung cancer. Recently, generative AI (gAI) technologies like ChatGPT have started to revolutionize medical education. ChatGPT is increasingly used to support medical students in learning complex concepts, developing research questions, and exploring clinical decision making through virtual patient simulations [58]. The role of ChatGPT in medical education has proven to be transformative. According to recent studies, generative AI can generate medical case studies, support clinical decision making by expanding differential diagnosis options, and assist in problem-based learning (PbL) scenarios [58].

Furthermore, AI has been shown to be a powerful tool for creating interactive and adaptive learning environments where students can engage in real-time questioning and receive immediate feedback, helping them to refine their clinical reasoning skills. In assessments, ChatGPT has also been experimented with creating multiple-choice questions (MCQs) and enhancing virtual patient simulations, making medical training more interactive and comprehensive [58]. Despite these advances, the adoption of AI in medicine still faces major challenges, including the need for clinical validation, integration into hospital workflows, and the management of data privacy and security [56]. However, its potential to revolutionize healthcare is undeniable, promising faster and more accurate diagnoses, personalized treatments, and greater efficiency in healthcare systems.

## 3. AI in Hematology

AI is reshaping the field of hematology by introducing cutting-edge approaches to address persistent challenges in diagnosing, classifying, and managing blood-related diseases. Conditions in hematology—ranging from simple cytopenias to complex malignancies like leukemia, lymphoma, and myelodysplastic syndromes—demand the synthesis of a wide array of information, including morphological evaluations, immunophenotypic profiles, cytogenetic data, molecular analyses, and clinical observations. Historically, the interpretation of these diverse data sources has depended on the expertise of clinicians and labor-intensive manual methods, which are susceptible to variability and often struggle to manage the volume and complexity of modern datasets. AI introduces a significant shift in this context, offering the capability to automate the integration and analysis of high-dimensional data, thereby boosting diagnostic accuracy and streamlining laboratory workflows. Within this landscape, machine learning and deep learning—two core components of AI—have become integral to hematologic applications. These technologies are used to detect patterns in medical imaging, such as blood and bone marrow smears, differentiate cellular populations in flow cytometry, and forecast disease evolution using genetic and clinical variables [59,60,61,62,63,64].

For example, convolutional neural networks (CNNs) have demonstrated high performance in identifying leukemic blasts, while algorithms such as support vector machines (SVMs) and random forests are used to predict patient outcomes and treatment responses based on molecular data. AI is also valuable in detecting subtle cellular or morphological features that may not be evident to human experts, particularly in early or diagnostically uncertain cases [65].

Beyond diagnostics, AI contributes to multiple aspects of patient management, including risk assessment, detection of minimal residual disease, and predictions of therapeutic efficacy. In research, AI accelerates the discovery of new biomarkers and enhances disease subclassification through the analysis of extensive omics datasets. As advancements in computing capabilities and algorithmic development continue, the adoption of AI in clinical hematology is transitioning from a theoretical possibility to an essential tool. Nevertheless, several hurdles—such as the need for standardized data, robust validation methods, interpretability of models, and seamless clinical integration—still need to be addressed. Despite these ongoing challenges, AI stands out as a powerful ally in transforming complex data into actionable clinical insights, paving the way for more precise, individualized, and efficient hematologic care [66,67,68,69,70].

## 4. AI in Hemophilia

Despite its rarity, hemophilia presents a unique test case for AI applications due to the availability of genetic markers, defined treatment protocols, and the increasing adoption of digital health tools. Both hereditary hemophilia (A and B) and acquired hemophilia pose diagnostic and management challenges that AI can potentially address through advanced pattern recognition, risk prediction, and imaging interpretation.

### 4.1. Diagnostic Applications of AI in Hemophilia

Artificial intelligence has already demonstrated significant utility in improving diagnostic workflows across various hematologic diseases, and its application in hemophilia is beginning to show promising results. For example, machine learning can analyze large volumes of data to identify complex patterns that are difficult for rule-based systems and human experts to detect. Its use in laboratory medicine is particularly promising, as laboratory tests form a crucial foundation for clinical decision making. Although still in its early stages, machine learning has already been employed to automate laboratory tasks, enhance resource utilization, and develop personalized reference ranges and test interpretations. The existing literature suggests that machine learning will become increasingly important for laboratory professionals. It is expected that future laboratories will adopt these techniques to achieve significant improvements in efficiency and diagnostic accuracy [71].

A systematic review conducted an analysis of the application of digital tools in the specific context of hereditary coagulation disorders. The literature search yielded a total of 21 relevant publications. Of these, 12 focused explicitly on artificial intelligence technologies, highlighting their growing relevance and potential in this field [72,73,74,75,76,77,78,79,80,81,82,83], while the remaining nine explored various other digital applications [84,85,86,87,88,89,90,91,92]. The integration of digital solutions into the healthcare management of hereditary coagulation disorders represents a significant advancement, offering promising opportunities to improve patient outcomes. In particular, AI-driven approaches hold considerable potential to enhance diagnostic accuracy, enable more precise prognostic assessments, and support the development of personalized therapeutic strategies. However, it is important to recognize that most of the current research remains in its early stages and is primarily based on retrospective analyses. Strategies such as transfer learning, synthetic data generation, and multi-center collaborations could help address the limitations posed by small datasets in hemophilia AI. Moreover, common challenges such as overfitting and reduced generalizability to new patients—especially in rare diseases—must be carefully managed through robust validation approaches [66,67,68,69,70]. As the field progresses, prospective studies and rigorous clinical validation will be essential to fully realize the benefits of these innovative digital tools. For widespread clinical adoption, large-scale studies, regulatory certifications as medical devices, and broader implementation across healthcare settings will be necessary elements that were notably absent from the reviewed literature. Moreover, the studies focused on digital applications primarily described the development and implementation of software aimed at improving patient care. These included telemedicine platforms and mobile health applications designed to facilitate disease management and enhance patient engagement. Of the nine studies, four employed a prospective design and included specific interventions [86,88,89,90]. For example, videoconferencing was used to support patients experiencing acute bleeding episodes [88]. One study employed a quality-of-life questionnaire to longitudinally assess patient burden, which could inform treatment decisions [89]. Another compared audio and video conferencing to evaluate their effectiveness in improving treatment planning [86]. Additionally, the use of a chatbot was explored to enhance patient education and self-management capabilities [90]. While these interventions were predominantly professional-led through digital platforms, true patient-centered applications based on AI or AI-generated recommendations remain rare. For instance, AI could be used to identify patterns indicative of potential disorders within patient data prior to laboratory testing, thereby helping to select individuals who require further investigation. Furthermore, AI could assist in personalizing treatment regimens, such as dosage adjustments tailored to specific clinical scenarios or behavioral coaching. Similar applications are already in use in other medical fields, including cardiology, oncology, and pain management [91,93].

Accurate and early diagnosis of hemophilia is crucial for preventing complications such as joint damage and for guiding optimal therapy initiation. In hereditary hemophilia, diagnosis traditionally relies on a combination of family history, coagulation assays, and genetic testing. In acquired hemophilia, which often presents nonspecific bleeding and lacks a hereditary pattern, diagnosis can be delayed or missed altogether. AI systems offer an opportunity to streamline this process by integrating heterogeneous data sources—such as clinical symptoms, laboratory values (e.g., factor activity levels, aPTT), and even imaging findings—to support more rapid and precise diagnostic conclusions. Recent advancements highlight how AI algorithms can support or even surpass traditional diagnostic approaches. For example, a study used supervised ML methods to categorize the severity of hemophilia A by analyzing both phenotypic and genotypic information, such as mutations in the F8 gene [94]. Traditionally, hemophilia A is considered an X-linked disorder predominantly affecting males, with females often being asymptomatic carriers. However, this study highlighted that certain F8 variants in females can lead to clinically significant bleeding disorders, which are frequently underdiagnosed [94]. By utilizing multiple datasets of F8 variants and associated disease severities, the researchers trained ML models that achieved high predictive performance, with F1 scores ranging from 0.88 to 0.99 across validation sets. This approach highlights the potential of AI in uncovering and accurately classifying the severity of hemophilia A in historically overlooked populations, thereby facilitating timely and appropriate clinical interventions. This classification is essential not only for predicting outcomes but also for guiding prophylactic treatment dosages [94].

Likewise, another study showed how deep learning techniques could identify new genetic interactions and modifier genes related to coagulation factor deficiencies, offering a more comprehensive molecular framework for diagnosing both common and rare bleeding disorders [95]. The research was confined to the PubMed database, selecting publications that mentioned at least one of the following terms: “genetics,” “genomics,” “genetic,” “mutation(s),” “polymorphism(s),” or “genetic variation.” This secondary query yielded 34 results, which were manually reviewed to assess their relevance, resulting in a final selection of seven papers, five of which were authored by the same research group [75,96,97,98,99,100,101]. A notable observation is that only a limited number of coagulation factors and their associated inherited bleeding disorders have been extensively analyzed. Specifically, five studies concentrated on FVIII and hemophilia A (HA)-causing variants [75,96,97,98,99], while the remaining two focused on factor IX (FIX)/hemophilia B and factor V (FV)/parahemophilia (i.e., FV deficiency), respectively [100,101]. This is not particularly surprising, considering that hemophilia A and B are among the most common inherited bleeding disorders, providing sufficient data to facilitate ML applications. Although FV deficiency is rarer, the structural similarities between FV and FVIII proteins justify its inclusion. Most of the retrieved studies share a common objective: predicting the structural and functional impact of genetic variants and establishing genotype–phenotype correlations to forecast disease severity. Despite employing different data collection strategies and ML approaches, their aims remain aligned. A particular study investigates how mutations in the FVIII protein influence the severity of HA [97]. The authors analyzed the structural properties of FVIII using a dataset of 443 missense variants across 364 positions (164 severe, 202 mild, and 77 moderate), all obtained from the European Association for Haemophilia and Allied Disorders F8 database. Data on surface exposure, hydrophobicity, and torsion angles were extracted from the FVIII structure (Protein Data Bank accession: 2R7E) and correlated with reported clinical phenotypes. As anticipated, mutations occurring at conserved, buried residues with low solvent accessibility were associated with severe HA phenotypes, likely due to disruption of the protein’s core structure, thereby impairing stability and function. Additionally, a residue interaction network for FVIII was constructed, where each amino acid served as a node, and connections represented proximity. Central residues within this network—characterized by high connectivity and low constraint—were more frequently linked to severe HA when mutated. Traditionally, the assessment of hemophilia severity relied on direct genotype–phenotype correlations using limited statistical approaches to interpret specific mutations. However, these methods were often insensitive to rare variants or those with complex structural effects. To overcome these limitations, the researchers developed an ML classifier named Hema-Class, trained on genetic, structural, and clinical data from the selected variants. Six ML models were evaluated, achieving accuracy ranging from 66% to 87%. The classifier was validated through in vitro alanine mutation screenings of the FVIII A2 and C2 domains, confirming its capacity to predict mutation severity. Hema-Class was subsequently employed to predict the effects of all possible FVIII missense variants, including those not yet observed. The model successfully identified regions where amino acid substitutions are more likely to result in severe HA symptoms [97]. This study underscores several critical steps necessary for developing accurate and robust ML models in the context of genetic variant analysis. First, it is essential to base models on carefully curated datasets that integrate both genetic and clinical information from patients. Leveraging multiple databases can ensure comprehensive coverage and facilitate the creation of separate training and validation sets, thereby minimizing bias and overfitting. Second, the selection of variants for analysis is crucial. Currently, most studies focus solely on missense variants, often explicitly excluding those associated with cross-reactive material positive cases. A more comprehensive approach would encompass various variant types, including nonsense mutations, in-frame and out-of-frame insertions/deletions, and splicing variants. In all cases, incorporating protein structural information and functional data—derived from in vitro experiments or in silico predictions—is vital for understanding the impact of specific variants on protein activity [95].

One of the most compelling diagnostic innovations involves the use of AI in medical imaging. Traditional joint assessments are limited by operator variability and the need for specialized musculoskeletal radiologists, whereas AI-enhanced imaging provides standardized, reproducible, and potentially point-of-care solutions. Based on this consideration, a study developed an AI-assisted ultrasound platform that detects hemarthrosis and synovitis—common but often underdiagnosed complications in people with hemophilia [102]. In particular, the researchers, using a convolutional neural network (CNN), developed and evaluated an AI algorithm trained on 3435 ultrasound images of elbow, knee, and ankle joints from individuals with hemophilia. The AI model demonstrated high diagnostic performance, achieving AUC values of ≥0.87 for hemarthrosis detection and ≥0.90 for synovitis detection. Specifically, the AI system identified hemarthrosis in 87% of elbow images, 80% of knee images, and 80% of ankle images where no bleeding was documented in the patients’ medical records. Similarly, synovitis was detected in 84% of elbow images, 75% of knee images, and 84% of ankle images without prior clinical indications. These findings suggest that AI-assisted ultrasound imaging can identify subclinical joint bleeding and inflammation, enabling earlier interventions and potentially preventing long-term joint damage. The integration of such AI tools into clinical practice could improve diagnostic accuracy and accessibility, particularly in settings lacking specialized radiological expertise [102].

Moreover, the diagnostic potential of AI may be extended further through integration with digital pathology and electronic health record (EHR) systems. Digital pathology, in particular, provides a robust platform for the application of AI in clinical diagnostics, enabling high-throughput image analysis and pattern recognition at a level of precision and reproducibility unattainable by human assessment alone [103]. When coupled with AI algorithms, whole-slide imaging can be leveraged to detect subtle histopathologic changes that may be overlooked in traditional microscopy, thereby enhancing the early detection of abnormalities. In translational medicine, such integration has already shown promise in refining disease stratification, prognostic modeling, and response prediction [104]. These capabilities are especially valuable in hematologic disorders, where morphological nuances can carry significant clinical implications.

In the context of acquired hemophilia, often misdiagnosed, AI systems may offer critical support in early detection by identifying atypical lab trends, such as isolated prolonged aPTT, and correlating them with patient histories captured in EHRs. In particular, a review highlighted that AI tools are increasingly capable of synthesizing complex datasets to flag potential bleeding risks or complications that may not be evident through conventional diagnostic workflows. This is particularly pertinent in acquired hemophilia, where delayed diagnosis can lead to severe outcomes. Therefore, the integration of AI into digital pathology and EHR systems could serve not only to enhance diagnostic accuracy but also to improve clinical vigilance for rare hematologic entities [105].

### 4.2. Prognostic Modeling and Risk Stratification

In hemophilia care, prognosis is shaped by a complex interplay of biological, clinical, behavioral, and therapeutic variables. The traditional tools used to assess risks such as bleeding scores, inhibitor development history, and pharmacokinetic profiles—are limited by their static nature and reliance on clinician experience. Artificial intelligence offers an advanced, data-driven alternative to prognostic modeling, capable of identifying subtle patterns in high-dimensional datasets that may elude conventional statistical analysis. One significant application of AI in this context is bleeding risk prediction. Ai et al. developed an ML model specifically to assess physical activity-related bleeding risk in children with hemophilia A. By incorporating physical activity logs, joint status, treatment history, and laboratory data, the model was able to generate personalized risk estimates that could be used to guide both clinicians and caregivers in adjusting activity levels or prophylactic regimens. Such dynamic risk tools are especially important in pediatric hemophilia, where balancing joint protection with healthy physical development is a constant challenge [76].

In addition to bleeding risk, AI has been proposed to predict long-term joint outcomes, quality of life metrics, and even psychosocial trajectories in people with PWH. Despite some initial limitations, these models can evolve over time through the reinforcement of learning frameworks. In particular, a study applied various AI and ML models to the American Thrombosis and Hemostasis Network (ATHN) dataset, aiming to predict poor outcomes in PWH. Although the models demonstrated higher accuracy and precision compared to traditional logistic regression, they showed limited recall rates—consistently below 53% -indicating a reduced ability to identify all individuals at risk. These findings highlight the need for larger and more comprehensive datasets, including longitudinal patient-reported outcomes and genotypic information, to enhance the predictive power of AI/ML models in hemophilia [97,106].

Reinforcement learning has gained traction in critical care settings, and similar architectures could be adapted to continuously update patient risk profiles as new clinical events occur. For example, a system might learn to recognize the patterns that precede inhibitor development or treatment failure, allowing clinicians to intervene earlier [107].

Moreover, unsupervised ML algorithms can cluster patients into risk subgroups without predefined labels, which may uncover novel phenotypes or treatment response categories within the hemophilia population. These tools are especially useful in small or heterogeneous datasets, which are typical in rare diseases like hemophilia [66].

### 4.3. Treatment Optimization and Clinical Decision Support

Treatment in hemophilia has become increasingly sophisticated, transitioning from episodic factor replacement to individualized prophylaxis and, more recently, to non-factor therapies and gene-based approaches. However, with the growing complexity of therapeutic options comes an urgent need for intelligent systems capable of optimizing treatment decisions based on individual patient characteristics. AI offers significant promise in this domain through its ability to analyze large and diverse datasets to derive actionable clinical insights. One of the primary uses of AI in treatment optimization is the modeling of pharmacokinetics (PK) for clotting factor concentrates and novel agents. Traditional PK modeling requires multiple blood drawings and complex calculations, often posing a barrier to routine use. AI can simplify and automate this process by predicting patient-specific PK profiles from limited input data, thus supporting the tailoring of infusion schedules. A study highlights how AI-enhanced PK platforms are facilitating the move toward “precision prophylaxis”, ensuring optimal factor levels are maintained while minimizing product waste and infusion burden [93].

Another critical application involves therapy selection. In related hematologic conditions such as multiple myeloma and amyloidosis, ML models can integrate genomic, clinical, and imaging data to recommend treatment regimens likely to result in better outcomes [60,61]. Translating this approach to hemophilia, AI systems can analyze patient-specific factors—such as inhibitor status, joint health, genetic polymorphisms, and lifestyle—to support clinical decisions between factor replacement therapies, bispecific antibodies (e.g., Emicizumab), or gene therapy candidates [108,109,110]. By integrating data from genetic sequencing, musculoskeletal imaging, treatment history, and even real-time wearable devices, AI models can assist clinicians in tailoring therapeutic strategies that reflect both clinical efficacy and patient preferences.

For instance, in patients with high-titer inhibitors, AI algorithms may identify early those who are poor responders to conventional replacement therapies and suggest the early use of non-factor options such as Emicizumab. Clinical studies have shown that Emicizumab prophylaxis—administered subcutaneously once a week or biweekly—significantly reduces bleeding rates compared to no prophylaxis in people with hemophilia A without inhibitors [111].

Similarly, ML can be used to stratify patients based on their eligibility or expected response to gene therapy, considering factors such as liver function, compatibility with viral serotypes, and the risk of immune responses. For example, gene therapy using the AAV5-hFVIII-SQ vector has demonstrated sustained clinical benefits in people with hemophilia A, with a substantial reduction in annualized bleeding rates and the complete discontinuation of prophylactic factor VIII use among those receiving therapeutic doses [112].

Moreover, AI can support longitudinal monitoring by detecting early signs of joint deterioration through automated image recognition or by identifying therapy adherence issues using data from digital health tools. These dynamic, personalized care models could not only optimize clinical outcomes but also improve quality of life by reducing the treatment burden and the risk of complications. Ultimately, AI-driven decision support may play a pivotal role in advancing precision medicine in hemophilia care, shifting the paradigm from reactive management to proactive, predictive, and individualized strategies [113].

### 4.4. Monitoring, Rehabilitation, and Long-Term Care

The successful management of hemophilia does not end with the administration of clotting factors or biologics but rather requires a lifelong, multidisciplinary approach encompassing joint health, physical activity, mental well-being, and social integration. Artificial intelligence is uniquely positioned to enhance these broader aspects of care through continuous monitoring, remote assessment tools, and intelligent rehabilitation strategies. A study has indeed examined how AI can assist in designing and optimizing rehabilitation programs for individuals with hemophilia, especially those with pre-existing joint damage [114]. By analyzing movement data from wearable sensors, AI models can evaluate joint function, detect gait abnormalities, and track progress over time. This supports a shift toward outcome-based physiotherapy, where interventions are tailored and dynamically adjusted based on patient response rather than a one-size-fits-all protocol [114]. In the psychosocial domain, AI models could eventually predict adherence risks or flag signs of depression and anxiety in adolescents with hemophilia, supporting timely psychosocial interventions. Integration with school or workplace accommodations, facilitated by digital health records, could also be envisioned in a future ecosystem where AI supports not just clinical outcomes but holistic patient well-being [115,116,117] (Table 1).

## 5. Ethical and Regulatory Considerations

Artificial intelligence applications in hemophilia raise a number of critical ethical and regulatory challenges that must be addressed to ensure safe and equitable deployment in clinical settings. One of the foremost concerns is the presence of bias in AI models, often resulting from training datasets that underrepresent certain populations, such as women or ethnic minorities. These biases can lead to skewed predictions, diagnostic disparities, and the amplification of existing healthcare inequalities [118].

Transparency and explainability are equally essential. Many AI systems function as “black boxes,” providing little insight into how decisions are made—an unacceptable limitation in clinical care where accountability is vital. The development of interpretable models or the use of explainability frameworks is critical to ensure that healthcare professionals can understand, trust, and appropriately act upon AI-generated outputs.

Patient consent and data privacy are also central to the ethical use of AI in medicine. Because AI systems rely on vast and often sensitive datasets, there must be strict adherence to data protection frameworks such as the General Data Protection Regulation (GDPR). Patients should be fully informed about how their data are collected, used, stored, and shared, especially when contributing to training or validating AI models. Anonymization and robust cybersecurity practices are essential to mitigate privacy risks [119].

Finally, before AI tools can be integrated into routine care, they must undergo thorough clinical validation to demonstrate their safety, effectiveness, and generalizability. This process must be accompanied by clear regulatory oversight at national and international levels, with standards adapted to the unique challenges of machine learning technologies [120]. Collaboration among clinicians, data scientists, developers, and policymakers is vital to ensure that these innovations align with ethical principles and truly serve patient interests [121].

## 6. Conclusions

The integration of AI into the diagnosis, management, and treatment of hemophilia marks a transformative step toward precision medicine in rare bleeding disorders. Through advanced machine learning models and data analytics, AI facilitates early diagnosis, improves patient stratification, and enhances decision-making processes for personalized treatment plans. The capacity of AI to analyze vast and complex datasets allows for the identification of disease patterns that are often undetectable through traditional methods, thereby reducing diagnostic delays and optimizing therapeutic outcomes. Although most applications of artificial intelligence in hemophilia are still under investigation and not yet adopted in routine clinical practice, preliminary findings suggest that these tools hold significant potential to support real-world patient management, particularly by enhancing diagnostic accuracy, individualizing treatment strategies, and improving overall care efficiency [8,9,63].

Moreover, AI-driven technologies are revolutionizing imaging diagnostics for hemophilia-related complications, such as joint damage and hemarthrosis, enabling real-time assessments and more accurate monitoring of disease progression [66,67]. This not only enhances patient quality of life but also reduces long-term medical costs by preventing severe complications through early intervention [7,18].

The promise of AI extends beyond current clinical applications, paving the way for innovative therapies, predictive analytics, and even gene therapy optimization in the near future [64,65,93]. As digital health tools continue to evolve, the seamless integration of AI into clinical practice holds the potential to redefine the standard of care for individuals with hemophilia, ensuring a more efficient, effective, and patient-centered approach [10,19].

To fully realize these benefits, it is essential to address several ethical and regulatory challenges. These include potential bias in training datasets, issues of data privacy and patient consent, and the need for transparent, explainable models in clinical decision making [118,119,120,121].

In summary, AI represents not just technological advancement but a paradigm shift in hemophilia care, offering a glimpse into a future where technology and medicine work in tandem to enhance patient outcomes and transform the management of bleeding disorders [69,93]. However, to fully harness the benefits of AI, ongoing efforts are required to address challenges related to data standardization, privacy concerns, and clinical validation [8,9,50]. Collaborative efforts among researchers, clinicians, and technology developers are crucial to ensure that AI-driven solutions are both safe and ethically implemented [8,9].

## Figures and Tables

**Table 1:** AI application in hemophilia.

Application	AI Technology Used	Clinical Impact	Status of Implementation	
Early Diagnosis	Supervised machine learning (e.g., classification algorithms) for genotype–phenotype correlation and severity prediction	Timely identification of hemophilia A and B severity, including in underdiagnosed female carriers	Still in research	[94,97]
Risk Prediction	Predictive analytics, machine learning models	Identification of bleeding risks and inhibitor development	Still in research	[76]
Imaging Diagnostics	Convolutional neural networks (CNNs) for AI-assisted ultrasound	Enhanced detection of joint damage and synovitis	Still in research	[102]
Prognostic Modeling	Supervised learning, Reinforcement learning	Prediction of disease progression and joint outcomes	Still in research	[66,67,68,69,70,106]
Treatment Optimization	Pharmacokinetics modeling, data analysis	Precision in therapy adjustments and dosage planning	Still in research	[93,112]
Monitoring and Long-Term Care	Wearable sensors, digital health tools	Improved patient monitoring and rehabilitation	Still in research	[114,115,116,117]

## Data Availability

Not applicable.
